# Mind Your Language: New Insights on Memory and Cognitive Aging Research Through Real-Life Methods

**DOI:** 10.1093/geroni/igaa057.2122

**Published:** 2020-12-16

**Authors:** Maximilian Haas, Alexandra Hering, Matthias Kliegel

**Affiliations:** 1 University of Geneva, Geneva, Switzerland; 2 University of Geneva, Geneva, Geneve, Switzerland

## Abstract

In the past decades, the so-called “age - prospective memory paradox”– a phenomenon comparing prospective memory (PM) performance in and outside the lab – has challenged the classical assumption that older adults necessarily evidence a marked decline in PM functioning. In our study, we want to extend established methods for measuring memory through arising technologies, such as the Electronically Activated Recorder (EAR; Mehl, 2017). Over the course of three days, 60 younger adults (18-32 years) and 45 older adults (60-82 years) completed an ambulatory assessment with the EAR in order to detect spontaneous speech production related to memory and memory failures. Results reveal that younger and older adults do not differ in the total number of utterances related to different facets of memory and cognition. However, when it comes to failures, older adults talk significantly less about PM failures than younger adults. Possible explanations for these findings will be discussed.

